# Oxidative stress and motion sickness in one crew during competitive offshore sailing

**DOI:** 10.1038/s41598-022-05219-6

**Published:** 2022-01-21

**Authors:** Tommaso Antonio Giacon, Gerardo Bosco, Alessandra Vezzoli, Cinzia Dellanoce, Danilo Cialoni, Matteo Paganini, Simona Mrakic-Sposta

**Affiliations:** 1 Department of Biomedical Sciences, Environmental and Respiratory Physiology, University of Padova, Via Marzolo 3, 35131 Padua, Italy; 2 Institute of Clinical Physiology, National Research Council (CNR), Milan, Italy

**Keywords:** Physiology, Biomarkers, Medical research, Molecular medicine

## Abstract

Competitive Offshore Ocean Sailing is a highly demanding activity in which subjects are exposed to psychophysical stressors for a long time. To better define the physiological adaptations, we investigated the stress response of subjects exposed to 3-days long ocean navigation with disruption of circadian rhythms. 6 male subjects were involved in the study and provided urine and saliva samples before setting sail, during a single day of inshore sailing, during 3-days long ocean navigation, and at the arrival, to measure oxidative stress, cortisol, nitric oxide metabolites (NOx) and metabolic response. Motion Sickness questionnaires were also administered during the navigation. The crew suffered a mean weight loss of 1.58 kg. After the long navigation, a significant increase in ROS production and decrease in total antioxidant capacity and uric acid levels were observed. Lipid peroxidation, NO metabolites, ketones, creatinine, and neopterin levels were also increased. Furthermore, a significant increase in cortisol levels was measured. Finally, we found a correlation between motion sickness questionnaires with the increase of NOx, and no correlation with cortisol levels. Physical and psychological stress response derived from offshore sailing resulted in increased oxidative stress, nitric oxide metabolites, and cortisol levels, unbalanced redox status, transient renal function impairment, and ketosis. A direct correlation between motion sickness symptoms evaluated through questionnaires and NOx levels was also found.

## Introduction

Sailing is a worldwide popular activity that includes various types of boats and disciplines. Offshore Ocean Sailing (OOS) could represent a relaxing leisure activity, but in case of competitions and regattas it is considered one of the most extreme endurance sports, exposing the crew to long-lasting, physically and psychologically demanding efforts^1^. Competitive OOS usually implies prolonged periods—ranging from days to months—spent at sea, in an extremely harsh environment, in isolation and self-sufficiency, far from safe harbours and with limited access to external aid or rescue^2^. The boat could represent an extremely uncomfortable, cold, wet, unstable, and enclosed environment without any privacy or comfort. In particular, racing boats are performance-oriented and with little or no comfort onboard. Energy expenditure during offshore sailing is high^3^, and it is challenging to maintain an adequate nutrient intake onboard, especially during harsh weather conditions^4^. Negative energy balance often results in weight loss, decreased body fat percentage, and reduced muscle strength, proportionally to the length of the race^5,6^. Proper sleep management is also essential to maintain adequate performance^7^. Sailors adopt polyphasic sleep techniques and incur severe sleep restrictions during competition, thus resulting in cognitive performance and alertness decrease^8^.

The study of adaptations to extreme environments is gaining popularity. Nonetheless, the literature exploring short- and medium-term adaptations to OOS is still insufficient. Seafarers are also exposed to high and prolonged stress levels. Loneliness, circadian rhythms disruption, and fatigue often result in alterations in their physical^9^ and psychological domains^10^. Consistently, high-stress levels can induce a modification in normal circadian fluctuations of cortisol, a glucocorticoid whose peak level in normal conditions is recorded after awakening^11^. During OOS and other highly stressing activities, a flattening of this curve has been recorded, with sustained high cortisol levels throughout the effort^12^. Due to the constant instability, the maritime environment significantly impacts cognitive and neuromuscular activity^13^. Moreover, motion sickness often affects people exposed to transportation and visual instability. The underlying cause is still ambiguous, some theories address a possible sensory mismatch mechanism between perceived and expected stimuli^14^. Others ascribe it to situations in which we are not able to maintain postural stability such as during maritime navigations^15^. A large percentage of people experience seasickness, with higher work-related risks and detrimental effects on the performance of sailors and seafarers^14,16^.

During inshore and offshore sailing, physical effort is inconstant, characterized by high intensity and anaerobic bursts, with increases in oxygen consumption and heart rate^1,17–19^. Such activity often leads to heat loss and dehydration^5,20^, and produces muscle damage and oxidative stress (OxS). OxS levels have been investigated in other endurance sports, such as triathlon^21^, ultra-endurance races^22^, and swimming^23^, revealing an overproduction of Reactive Oxygen Species (ROS) and a depletion of total antioxidant capacity (TAC). The redox status—namely, the equilibrium between ROS and TAC—deeply affects intracellular function. Maintaining ROS homeostasis is crucial for normal cellular responses, while overproduction is deleterious and can damage cell structures (i.e., proteins, membrane, DNA), leading to progressive organism’s disfunction^24,25^. Along with ROS production, increased levels of nitrogen metabolites and in particular nitric oxide (NO), a crucial messenger in many tissues such as endothelium and gastrointestinal tissues, can be found under stressful condition^26^. Nonetheless, a formal assessment in sailing sports and specifically during OOS is still lacking.

This study aimed to investigate oxidative stress variations in sailors involved in OOS. To have a more accurate definition of stress, we also evaluated cortisol levels, biochemical profile, and renal function markers creatinine and neopterin. Motion sickness has been investigated through neurophysiological symptoms questionnaires.

## Methods

### Experimental design

**Figure 1:**
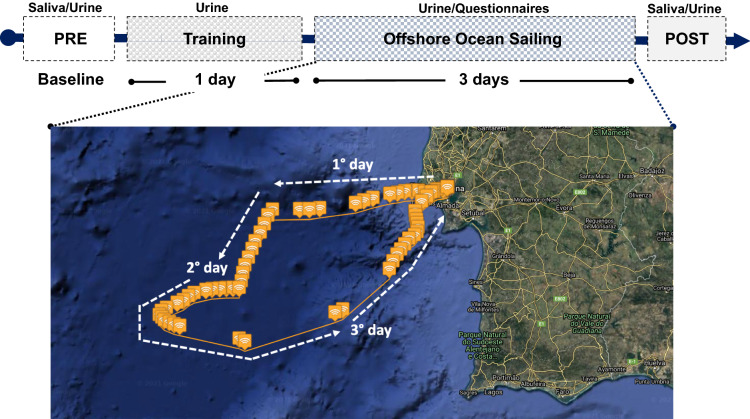
Sketch of the experimental protocol and map showing the navigation route. Data were collected before departure (PRE), during the inshore navigation (Training) during each day of Offshore Ocean Sailing (from 1° to 3° day), and at arrival (POST). GPS data and map were obtained with Spot Gen3, SPOT LCC, Globastar, Inc, Covington, Louisiana, USA. Maps by Google, Inst. Geogr. Nacional. Modified geographic map was edited with Microsoft Office PowerPoint 2007, Microsoft Corporation, Washington, USA, version 16.16.27, https://www.microsoft.com/it-it/microsoft-365/powerpoint and at the top was insert the experimental protocol timeline.

This observational study was carried on in November 2020 during an OOS racing-oriented training that included a theoretical part ashore, a full training day of inshore sailing, and 3 days of non-stop OOS roughly between the latitudes of Gibraltar and Lisbon. The crew sailed in a Class 40 (ITA 84) racing yacht and, during the navigation, was divided into two groups alternating rest and duty shifts every 3 h. Figure 1 depicts the study protocol and samplings. Urine and saliva samples and anthropometric measurements were obtained ashore during the theoretical part (PRE) and approximately two hours after the end of the navigation (POST) to be as accurate as possible. Further, two urine samples were obtained during the single day of inshore navigation (Training) and three times a day during OOS (Sailing).

### Subjects

This study involved six male sailors: the skipper (S.F.)—a professional sailor with experience in ocean solo races—and five recreational sailors with good expertise in seamanship The total number of subjects was determined by the maximum crew that the boat could support and by chance all the participants to this training were healthy male. Their characteristics are reported in Table 1.

**Table 1:** Anthropometric parameters of the six sailors.

	Age	Height (cm)	Weight (kg) PRE	Weight (kg) POST	BMI (kg m^−2^) PRE	BMI (kg m^−2^) POST
1	25	170	65.2	63	22.6	21.8
2	41	180	86.2	84.5	26.6	26.0
3	31	185	93.3	90.2	27.2	26.4
4	51	187	125.2	123.1	35.8	35.2
5	36	184	83.6	83.5	24.6	24.6
6	26	177	84.2	83.1	26.9	26.5
Mean ± SD	35.16 ± 9.70	180.50 ± 6.28	89.62 ± 19.76	87.90 ± 19.59*	27.28 ± 4.52	26.75 ± 4.50

Parameters collected from the sailors at Pre and Post.

*BMI* Body Mass Index. *p < 0.05.

### Navigation

The offshore navigation lasted 3 days, during which the crew sailed into the ocean for a total of 420 miles, with a top speed of 14.89 kn.

During the first day and night, the atmospheric conditions were challenging. Swell of 3/3.5 m Significant Wave Height (SWH) from NW and wind from 5 to 15 kn from S-SE resulted in a boat's inconvenient motion. During the day, the wind increased between 25 and 40 kn in gusts as sailors encountered two significant squalls and had to flee downwind. During the night, sailors were forced to maintain a 70° true wind angle (TWA) sailing upwind to cope with waves, and the wind speed increased up to 45 kn. After the first day, the crew was objectively stressed. These harsh conditions induced major seasickness and vomiting in one crew member, with a total inability to work on deck. This subject started to recover only at the end of the navigation, during which he never ate and vomited many times without being able to drink and rehydrate. Two other people vomited but were not impaired at work. Liquid reintegration started the day after. On the second day, the conditions changed, with waves height reduced to 1–2 m SWH. The boat headed downwind, hoisting a code zero sail, maintaining the boat flat at an average speed of 9–10 kn with 15–25 kn of wind speed. The navigation remained stable until the end of the navigation in Lisbon on the third day.

### Motion Sickness Questionnaire

To study motion sickness, previously validated Global Sickness Rating Scale (GSRS)^27^ and Motion Sickness Questionnaire (MSQ)^14^ were used. They have been administered once per day during the Offshore Sailing, in the evening, at 9 PM, according to the 3 h shifts.

### Saliva and urine collection

Approximately 1 mL of saliva was obtained before and after the training and collected in Salivette devices (Sarstedt, Nümbrecht, Germany) at 8 AM. The subjects were trained on the correct use as previously reported^28,29^.

Urine samples were collected by voluntary voiding in a sterile container before and after the training and every day during the training and navigation at 9 AM, 3 PM, and 9 PM according to the 3 h shifts. All samples were stored at 4 °C in a portable cooler on board and during the transport back to the laboratory. The specimens were then stored in multiple aliquots at − 20 °C until assayed and thawed only once before analysis.

### ROS and TAC

An X-band electron paramagnetic resonance spectroscopy (9.3 GHz) (E-Scan, Bruker Co., MA, USA) was used to detect ROS production and TAC values. Saliva samples were stabilized at 37 °C using a Temperature and Gas Controller “Bio III” unit (Noxigen Science Transfer & Diagnostics GmbH, Germany), interfaced with the E-Scan. ROS production and TAC assessment methods were previously described^28,30^. Samples were analyzed in triplicate.

### Cortisol

The concentration of free cortisol in the saliva was quantitatively determined through ELISA method according to the manufacturer’s protocol (COR(Cortisol) ELISA Kit; FineTest, Wuhan Fine Biotech Co.) as previously described^31^.

### 8-Isoprostane

Lipid peroxidation was assessed in urine by competitive immunoassay measuring 8-isoprostane concentration (8-iso-PGF2α) (Cayman Chemical, USA). The method was previously described^32^.

### NO metabolites

NO metabolites (NOx = NO_2_ + NO_3_) levels were assessed in urine by a method based on the Griess reaction^33^, using a commercial kit (Cayman, BertinPharma, Montigny le Bretonneux, France)^28,33^.

Every assessment was carried out in duplicate and read by a microplate reader spectrophotometer (Infinite M200, Tecan Group Ltd., Männedorf, Switzerland).

### Creatinine, neopterin, and uric acid

Urinary creatinine, neopterin, and uric acid concentrations were measured by isocratic high-pressure liquid chromatography. The calibration curves were linear over the range of 0.125–1 μmol/L, 0.625–20 mmol/L, and 1.25–10 mmol/L for neopterin, uric acid, and creatinine levels, respectively. Inter-assay and intra-assay coefficients of variation were < 5%. Methods were previously described^28,34^.

### Urine standard analysis

The Urine Test Strips (Combi screen 11sys PLUS, GIMA, Gessate, Milan, Italy) were used to semi-quantitative determinations of bilirubin, urobilinogen, ketones, proteins, blood, pH, leukocytes, and specific gravity/density in urine. The tests were performed in duplicate.

### Statistical analysis

Statistical analysis was performed using the GraphPad Prism package (GraphPad Prism 9.0.1, GraphPad Software Inc., San Diego, CA) and SPSS statistics software (IBM corporation). Data are presented as mean ± SD. Statistical analyses were performed using: non-parametric tests, Wilcoxon matched-pairs signed-rank test for independent samples (ROS and TAC and cortisol in saliva), due to the small sample size for compared pre vs. post and ANOVA repeated measures, with Dunn’s multiple comparison tests to further check the among-groups significance. p < 0.05 was considered statistically significant..dCohen was used for calculating the size effect, and Confidence Interval 95% for *d*_*Cohen*_ was calculated.

Change Δ% estimation [((post value-pre value)/pre-value) × 100] is also reported in the text. Spearman correlation (r) with 95% confidence intervals. Chi-square (χ^2^) test and phi coefficient (ϕ) were used to evaluate correlation and association.

### Ethics approval

This research study was approved by the Ethical Committee of University of Milan, Italy (Aut. n° 37/17). All procedures conformed to the standards set by the 1964 Declaration of Helsinki and its later amendments.

### Consent to participate

Informed consent was obtained from all individual participants included in the study.

### Consent for publication

All authors have read the manuscript and expressed their consent for the publication.

## Results

A significant difference (p < 0.05) was observed in weight (kg) between Pre and Post (Table 1). All crew members suffered a loss of weight (Mean weight loss: 1.58 kg).

**Figure 2:**
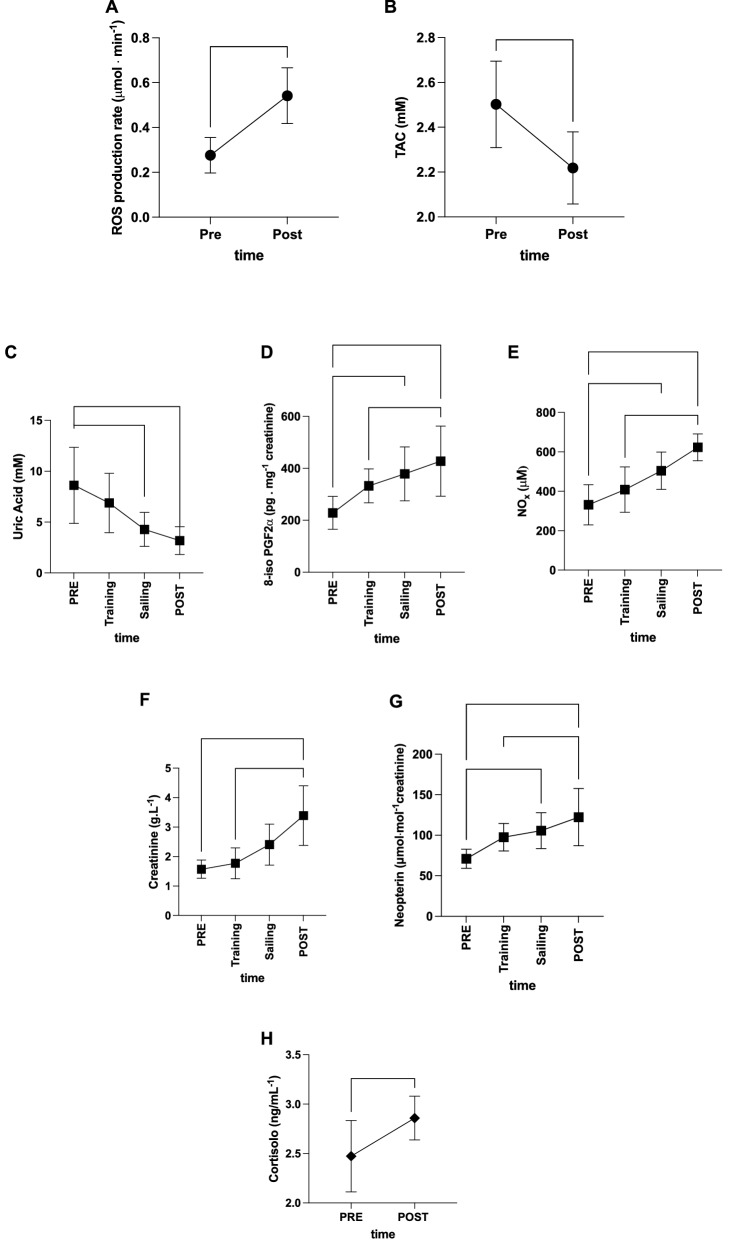
Biomarkers kinetic. Time course of: (**A**) radical oxygen species (ROS) production rate (μmol·min^−1^) and (**B**) total antioxidant capacity (TAC—mM) in saliva assessed by EPR; (**C**) uric acid (mM); (**D**) 8-isoprostane (8-iso-PGF2α, pg mg^−1^ creatinine); (**E**) nitric oxide metabolites (NOx, μM), (**F**) creatinine (g L^−1^), (**G**) neopterin (μmol mol^−1^creatinine), concentrations detected in urine. In (**H**) cortisol levels (ng/mL) measured in saliva. *p < 0.05, **p < 0.01, ***p < 0.001 significantly different. Figure created with: GraphPad Prism, GraphPad Software inc. California, USA, version 9.0.1, https://www.graphpad.com/.

An unbalance of oxidative stress was found. ROS production rate in saliva significantly (p < 0.01) increased at Post OOS (0.27 ± 0.07 vs 0.54 ± 0.16 μmol min^−1^, dCohen = 0.74; 95% CI 1.171–4.326; Fig. 2A) with a significant decrease (p < 0.05) in antioxidant capacity (TAC 2.50 ± 0.19 vs 2.21 ± 0.16 mM, dCohen = 1.65; 95% CI 2.961–0.341; Fig. 2B). In addition, uric acid measured in urines significantly decreased (range p < 0.05–0.001) during sailing (8.61 ± 3.74 vs. 4.28 ± 1.66 vs. 3.18 ± 1.36 mM at Post, dCohen = 1.49, 95% CI 2.777–0.216; and dCohen = 1.93, 95% CI 3.3–0.56 respectively; Fig. 2C). A significant increase (range p < 0.05–0.001) in lipid peroxidation during OOS (8-isoprostane 228.40 ± 63.1 vs 378.68 ± 103.69 pg mg^−1^ creatinine; dCohen = 1.75, 95% CI 0.42–3.081) and at Post OOS (8-isoprostane 427.70 ± 134.98 pg mg^−1^ creatinine, dCohen = 1.89, 95% CI 0.53–3.253; Fig. 2D) was measured; besides NO metabolites significantly (range p < 0.05–0.001) increased during OOS (NOx 331.8 ± 102.2 vs 504.5 ± 94.85 μM; dCohen = 1.75, 95% CI 0.422–3.085) and at Post (NOx 331.8 ± 102.2 vs 623.0 ± 68.24 μM; dCohen = 3.35, 95% CI 1.656–5.218) (Fig. 2E).

The time course in Fig. 2F,G showed a significant increase post OOS (p < 0.05–0.01) of creatinine (1.57 ± 0.31 vs. 1.77 ± 0.52 vs. 3.38 ± 1.01 g L^−1^; dCohen = 2.43, 95% CI 0.938–3.922) and neopterin/creatinine (70.92 ± 11.80 vs. 105.603 ± 22.14 vs. 23.33 ± 35.25 μmol mol^−1^ creatinine: dCohen = 1.99, 95% CI 0.609–3.377, and dCohen = 1.95, 95% CI 0.579–3.33) levels respectively at sailing and post OOS.

Finally, a significant increase (p < 0.05) in cortisol levels was measured Post OOS in saliva (2.47 ± 0.36 vs. 2.85 ± 0.22 ng/mL^−1^, dCohen = 1.27, 95% CI 0.033–2.515; Fig. 2H).

No significant differences were recorded in GSRS for different items and MSQ during the 3 days of offshore navigation (see Tables 2, 3).

**Table 2:** Global Sickness Rating Scale (GSRS), number of subjects and (total value) are reported for each day.

Sailors (n = 6)	Global Sickness Rating Scale (GSRS)		
Scores	Day 1	Day 2	Day 3
1: No symptoms	n2 (2)	n2 (2)	n5 (5)
2: Initial symptoms of motion sickness but no nausea	–	n1 (2)	–
3: Mild Nausea	n1 (3)	–	n1 (3)
4: Moderate Nausea	–	n1 (4)	–
5: Severe nausea and/or retching	–	n1 (5)	–
6: Vomiting	n3 (18)	n1 (6)	–
Total score	23 ± 3.8	19 ± 2.1	8 ± 0.8

Total scores ± SD are reported.

**Table 3:** Motion Sickness Questionnaire (MSQ).

Sailors (n = 6)	Motion Sickness Questionnaire (MSQ)		
	Day 1	Day 2	Day 3
General discomfort	1.0 ± 1.2	1.33 ± 1.03	0.83 ± 0.72
Fatigue	1.16 ± 0.98	1.16 ± 0.98	1.33 ± 0.81
Headache	0.83 ± 1.16	0.66 ± 1.21	0.33 ± 0.51
Eye Strain	0.16 ± 0.40	0.16 ± 0.40	0.33 ± 0.51
Difficulty focusing	0.16 ± 0.40	0.0 ± 0.0	0.16 ± 0.40
Increased salivation	0.0 ± 0.0	0.0 ± 0.0	0.16 ± 0.40
Fulness of head	0.80 ± 1.16	0.83 ± 0.51	0.33 ± 0.51
Blurred vision	0.0 ± 0.0	0.0 ± 0.0	0.0 ± 0.0
Dizziness as illusory sense of motion (eyes open)	0.33 ± 0.51	0.33 ± 0.51	0.0 ± 0.0
Dizziness as illusory sense of motion (eyes closed)	0.33 ± 0.81	0.66 ± 1. 21	0.16 ± 0.40
Vertigo	0.50 ± 0.83	1.33 ± 1.21	0.16 ± 0.40
Stomach awareness	1.50 ± 1.22	1.33 ± 1.21	0.83 ± 0.75
Burping	1.50 ± 1.22	0.66 ± 0.81	0.16 ± 0.40

Mean (± SD) values of the investigated variables.

Urine standard parameters are reported in Table 4. A significant increase in urinary ketones levels was detected during the navigation. pH and bilirubin values also increased but did not reach statistical significance.

**Table 4:** Urine standard analysis***.***

Sailors n = 6					
	PRE	Day 1	Day 2	Day 3	POST
Bilirubin (μmol L^−1^)	5.65 ± 8.80	6.16 ± 9.55	11.66 ± 9.07	17.00 ± 1.09	11.16 ± 8.75
Urobilinogen (μmol L^−1^)	0.2 ± 0.1	0.2 ± 0.1	0.2 ± 0.1	0.2 ± 0.1	0.2 ± 0.1
Ketones (mmol L^−1^)	1.0 ± 2.23	1.0 ± 2.0	16.16 ± 18.49*	12.16 ± 14.2*	9.0 ± 5.47*
Protein (mg dL^−1^)	0.0 ± 0.0	0.0 ± 0.0	0.0 ± 0.0	0.0 ± 0.0	0.0 ± 0.0
Blood (Ery μL^−1^)	0.0 ± 0.0	0.0 ± 0.0	0.0 ± 0.0	0.0 ± 0.0	0.0 ± 0.0
pH	5.16 ± 0.40	6.50 ± 1.25	6.12 ± 1.43	6.25 ± 1.04	5.87 ± 1.18
Leucocytes (Leuko μL^−1^)	0.0 ± 0.0	0.0 ± 0.0	0.0 ± 0.0	0.0 ± 0.0	0.0 ± 0.0
Specific gravity/density	1.01 ± 0.00	1.02 ± 0.00	1.03 ± 0.00*	1.03 ± 0.00*	1.03 ± 0.00*

Mean (± SD) values of the investigated variables in the urine test strip in the sailors. Changes in urine standard urinalysis referred to PRE are shown. Statistically significant difference at p < 0.05 (* symbol).

**Figure 3:**
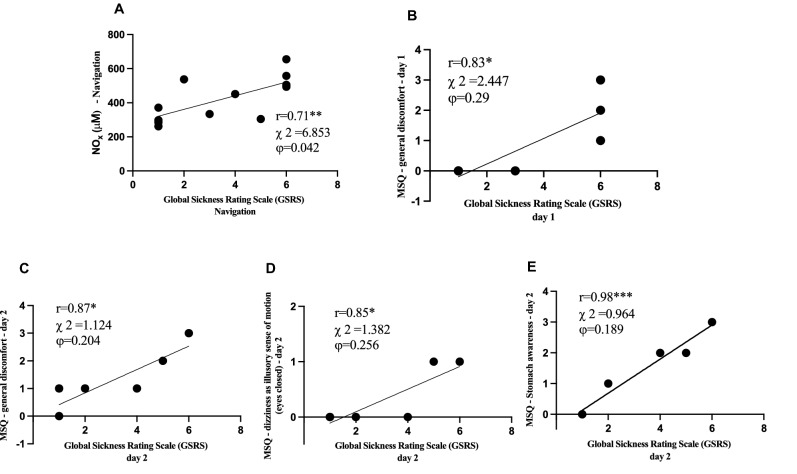
Relationship panel plot of: (**A**) NOx and GSRS during navigation; (**B**) GSRS and general discomfort of MSQ at 1st day; at the 2nd day, the panels show the correlation between GSRS and specific items of MSQ. (**C**) General discomfort and GSRS. (**D**) Dizziness as illusory sense of motion (eyes open) and GSRS. (**E**) Stomach awareness and GSRS. A significant linear relationship (p < 0.05–0.001) between parameters was estimated. The correlation coefficient (r), Chi-Square (χ^2^) and Phi coefficient (ϕ) are reported. Figure created with: GraphPad Prism, GraphPad Software inc. California, USA, version 9.0.1, https://www.graphpad.com/.

Finally, a positive relationship was found during navigation (training and sailing) between NO metabolites and Global Sickness Rating Scale (GSRS) scores (r = 0.94, p < 0.05) (Fig. 3A); between GSRS score and specific items of Motion Sickness Questionnaire (MSQ), in details: during the 1st day with General discomfort r = 0.83 (p = 0.04) (Fig. 3B), during 2nd day with General discomfort r = 0.87 (p = 0.02) (Fig. 3C), dizziness as illusory sense of motion (eyes open) r = 0.85 (p = 0.03) (Fig. 3D), and stomach awareness r = 0.98 (p = 0.0007) (Fig. 3E); during the 3rd day no correlation was found.

## Discussion

To our knowledge, this is the first study to investigate oxidative stress on urine and saliva sampled from non-professional sailors during OOS and possible correlation with motion sickness. This setting is particularly challenging, with rapid changes in terms of environmental conditions and circadian rhythms.

According to the results, subjects exposed to OOS suffer a significant multifactorial increase in oxidative stress and cortisol. A small number of studies considered modifications in cortisol levels in sailors and seafarers. Oldenburg et al. found that cortisol awakening levels were highly dependent on subjective stress perception and work type. Mental work was also associated with higher cortisol levels than physical work^35^. This is confirmed by cortisol levels found in maritime pilots, increasing their tasks' difficulty^36^. Some studies’ results reflect that seafarers’ cortisol levels are higher in port stays than at sea, probably because of the break of a working routine found during days at sea^35,37^. Liberzon et al. found that cortisol response at awakening in the crew increased with navigation time and was not correlated with sleep duration or patterns^37^. Other confined environments in which the crew suffers a sudden and prolonged change in circadian rhythms and psychosocial stress are, for example, military ships, submarines, or spaceships. In these environments, a flattening of the standard cortisol fluctuation profile has also been recorded under stressful conditions^38,39^. Similar results were obtained by Gunnarsson et al. on ocean sailors studied for a more extended period during an offshore regatta. They also reported an initial increase in cortisol levels at the beginning of the navigation, with a flattening of the fluctuation when sailors reached the regularization of the shift regimen^12^. Our study found a significant increase in cortisol levels in sailors after 3-day-long offshore navigation compared to their basal level (+ 15%, see Fig. 2H). These results may be related to the significant stressors that maritime personnel and offshore ocean sailors have to endure, particularly fatigue and poor sleep quality. From a physiological point of view, sleep disturbances induce a decrease in physical and cognitive performance in sailors^8^ and a disruption of normal cortisol secretion, causing the activation of pro-inflammatory pathways^40,41^. Physical exercise can also induce a modification in cortisol secretion^42^. During offshore sailing, a basal level of muscle activation is needed to cope with instability and to aid thermoregulation, but short bursts of anaerobic exercise are required in all the maneuvers^1,17–19^. It is, therefore, reasonable to think that these aspects also contribute to cortisol levels alterations.

During day one, three subjects suffered from motion sickness and had vomiting episodes. Although cortisol is known to correlate with acute nausea and vomiting^43,44^, we found no significant correlation between this hormone and the seasickness scale questionnaires administered^14,27^. Nonetheless, we have been able to measure nitric oxide metabolites (NOx) levels throughout the navigation. Nitric oxide is involved in many gastrointestinal mucosal mechanisms^26^, and previous studies found a correlation between salivary and serum NO levels with vomiting syndromes and Gastroesophageal Reflux Disease (GERD)^43–46^. Nausea caused by motion sickness is also characterized by gastric dysrhythmias^47^. In accordance with these studies, we found a significant linear relationship between NOx levels and GSRS during the first day of navigation, during which the subjects suffered the most intense motion sickness (Fig. 3).

During inshore regattas, short bursts of high-intensity activity are described^18,19,48^. However, even if data regarding physical effort during offshore sailing seem comparable with inshore activity^17^, the evidence is scarce, difficult to obtain, and limited to measuring the effects of energy expenditure and physical effort activity after the race. Weight loss, fat percentage decrease, lower limb strength, and muscle mass reduction are often reported^1,3,4,6,49^. ROS production is enhanced by exercise^21–23,29,50^. In particular, anaerobic exercise can induce prolonged oxidative stress up to 24 h after the effort^51,52^, which is then balanced by an enhanced antioxidant response^21,23,53^. In America’s Cup sailors, oxidative stress markers after the race were higher than their baseline levels, especially in crew members involved in high-intensity physical work^54^. Our study is the first to analyze oxidative stress markers during OOS. Our results show a significant increase in ROS production after the navigation. The imbalance between the ROS production rate (about + 100%) and the antioxidant scavenging (− 12%, see Fig. 2A–C) reflected the increase in the oxidative stress-related damage to lipids (+ 87%; see Fig. 2D). Oxidative stress is highly involved with inflammation and endothelial dysfunction in developing chronic cardiovascular diseases^55,56^. Even though more evidence should be produced on ocean sailors, the effects of oxidative stress exposition on seafarers can be a potential cause of their higher cardiovascular risk and mortality rate for coronary heart disease^57–59^.

Neopterin and creatinine concentration can increase during systemic oxidative stress, as shown in some studies^23,28,29,60^. Even if a decrease in kidney function can be a hint of organ damage during endurance sports, it is often the result of many physiological responses to stress and physical demands^21,22,61^. In our study, an increase of evaluated biomarkers concentrations was observed during and post-offshore sailing and was associated with ROS production. In any case, this study did not assess the chronic or long-term effects of offshore sailing. Mainly referred to kidney activity, the subjects manifested a temporary “impairment of renal function” as a likely physiological or adaptive response to dehydration. This could also be linked to significant weight loss (see Table 1) and vomiting, which changes ketones concentration, pH, and specific density (see Table 4). Ketones increase could also hint at how athletes’ metabolism copes with high energy demand and stress. Their production is stimulated by low insulin, high glucagon, and epinephrine concentrations, suggesting a shift to metabolic efficiency and fuel sparing of the organism exposed to endurance exercise and fasting^62,63^. They are also second messengers for many pathways, such as food intake stimuli^64^. The ketogenic regimen is also related to the increase of lipid metabolism^65^ which in the case of OOS is often associated with the decrease of body fat percentage and weight loss^4,6,49^. Considering that sailors are exposed to harsh environmental conditions and that motion sickness and working rates can influence nutrition habits during a race^4^, it is of utmost importance to maintain an adequate water intake during navigation to prevent renal damage and to keep proper caloric intake to sustain physical performance.

## Limitations and conclusions

As for other studies^4,6,12,17^ that focus on OOS, we found many difficulties in producing reliable data and scientific evidence. The researcher himself, which was part of the crew, had to take part in the strenuous activity schedule, the space for medical devices and samples on board is limited, invasive procedures are complicated to perform because of continuous motion, electronic devices cannot be charged because electrical power is limited and needed for navigation. The complexity of this environment often results in a lack of reliable literature^1^. Therefore, we chose to obtain urine and saliva samples because of the limited logistic disadvantages of these samples.

A limitation of this study is the lack of data on the quality and duration of sleep. This might have influenced the cortisol level, but Liberzon et al. found no connection between sleep and cortisol levels^37^, and other OOS experiments show data similar to ours^12^.

Some previous experiences in literature^66^ found a correlation between cortisol levels and motion sickness. We speculate that probably due to the low number of investigated subjects and saliva samples, and because we didn’t measure cortisol levels immediately after vomiting episodes, we found no significant correlation between motion sickness questionnaire results and cortisol levels. Therefore, to be more confident on these results our methods could be implemented. Another factor to be considered could be that we investigated only male subjects, and as previously reported by Meissner et al., the cortisol level changes in saliva in male patients could not be significant. Cortisol response in motion sickness, as they suggest, should be corrected for the hour of the day, gender, and basal cortisol levels^67^. Moreover, high variability was observed in oxidative stress markers, cortisol levels, and motion sickness scales between the same sailors on various days.

Another limitation is that we have not been able to obtain information on sailors’ cardiovascular and metabolic activity during the navigation, even though they have been described in other similar and comparable studies^17,18^. In the future, we hope we will be able to implement our methods and obtain this data in a similar environment.

However, the present offshore sailing study offers valuable information on the redox state, renal function, and motion sickness response during this high demanding activity. OOS has been shown to induce an increase in oxidative stress biomarkers and NO metabolites. A correlation was found also between the increase in NO metabolites level and motion sickness intensity evaluated through questionnaires and symptoms. In this experiment, a transient reduction in renal function was found. Moreover, salivary cortisol increased in response to physical activity and stress induced by navigation. Future studies are required to investigate the biochemical processes and the clinical correlations consequent to maritime exposure.

## Data Availability

The datasets generated and analyzed during the current study are available from the corresponding author on reasonable request.

## Abbreviations

8-iso-PGF2α: 8-Isoprostane
EPR: Electron paramagnetic resonance
GERD: Gastroesophageal reflux disease
GSRS: Global Sickness Rating Scale
MSQ: Motion Sickness Questionnaire
NO: Nitric oxide
NOx (NO_2_ + NO_3_): Nitric oxide metabolites
OOS: Offshore ocean sailing
OxS: Oxidative stress
ROS: Reactive oxygen species
SWH: Significant wave height
TAC: Total antioxidant capacity
TWA: True wind angle
